# Supplementary material for: Cyber-physical defense in the quantum Era
Source: Sci Rep. 2022 Feb 3;12:1905. doi: 10.1038/s41598-022-05690-1 (PMC8814167; doi:10.1038/s41598-022-05690-1)
Supplement: Supplementary file 1 — Supplementary Information 1. [file 41598_2022_5690_MOESM1_ESM.pdf]

Appendix A: Representative Cyber-physical Attacks Documented in the Media

**Espionage and sabotage of critical facilities**, such as US data breach in 2021 due to the SolarWinds attack or attempts of Saudi Aramco cyber-sabotage of oil-processing facilities in 2020. Similar problems are spanning worldwide.	**Adversarial actions in this scenario**, include USB injection of corrupted software binaries, drive-by-download malware installation, spear phishing-based design of websites, and traditional social engineering manipulation of critical infrastructure employees.
**Remote control of navigation systems**, including successful hacking of autonomous cars and avionic systems. Studies and general concern started with a malware that infected over sixty thousand computers of an Iranian nuclear facility.	**Adversarial actions** include the use of infection vectors (e.g., USB drives), corrupted updates and patches, radio frequency jamming, radio frequency spoofing, and software binary manipulations.
**Disruptions of large-scale industries** have been appointed by the Federal Office for Information Security of Germany as a serious concern to European factory and industrial markets. Similar threats affect drones and smart cities, as well.	**Adversarial actions** include the use of GNSS (Global Navigation Satellite Systems) attacks, e.g., jamming of signals, spoofing and hijacking of communications to downgrade communications to insecure modes (e.g., from encrypted to plain-text communications).
