# Supplementary material for: Cyber-physical defense in the quantum Era
Source: Sci Rep. 2022 Feb 3;12:1905. doi: 10.1038/s41598-022-05690-1 (PMC8814167; doi:10.1038/s41598-022-05690-1)
Supplement: Supplementary file 2 — Supplementary Information 2. [file 41598_2022_5690_MOESM2_ESM.pdf]

Appendix B: Quantum Computing Basics.

With quantum computing, the basic unit of information is the quantum bit or qubit. A qubit is a binary unit of data that is simultaneously a zero and a one until the end of its life when the qubit is measured, which ends either in state zero or one. There is a probability associated with each of these two outcomes. In the ket notation, a qubit is represented by the pair

$$q = a\,|0\rangle + b\,|1\rangle$$

The symbols $|0\rangle$ and $|1\rangle$, pronounced ket zero and ket one, denote the quantum states zero and one. The parameters a and b are called probability amplitudes. Raised to the power of two, i.e., a^2 and b^2, they respectively correspond to the probability of measuring the qubit in state zero or state one. The plus sign does not represent arithmetic addition. Rather, an expression with the plus sign should be interpreted as a superposition of its operands, in this case the quantum states $|0\rangle$ and $|1\rangle$. Superposition means that a qubit is both a zero and a one at the same time. Quantum computations are done by gates. For instance the Hadamard find many applications. It can calculate the arithmetic sum of the probability amplitudes $a+b$ and their difference $a-b$.

Several qubits can be grouped together to represent a complex problem. For instance, a two-qubit quantum state q_1q_0, where q_1 is equal to $a_1\,|0\rangle + b_1\,|1\rangle$ and q_0 is equal to $a_0\,|0\rangle + b_0\,|1\rangle$, corresponds to the superposition:

$$a_1a_0\,|00\rangle + a_1b_0\,|01\rangle + b_1a_0\,|10\rangle + b_1b_0\,|11\rangle$$

Interestingly, information can be contained in binary combinations but also in probability amplitudes of ket terms. Quantum machine learning leverages both forms of information representation.

Qubits may be entangled, that is, related together such that they read in a coherent way. This means that some of the reading outcomes are made more probable than others. Besides, some reading outcomes can be made entirely non-probable.
