# Supplementary material for: Cyber-physical defense in the quantum Era
Source: Sci Rep. 2022 Feb 3;12:1905. doi: 10.1038/s41598-022-05690-1 (PMC8814167; doi:10.1038/s41598-022-05690-1)
Supplement: Supplementary file 3 — Supplementary Information 3. [file 41598_2022_5690_MOESM3_ESM.pdf]

Appendix C: Bloch Sphere Representation of a Qubit

A qubit can be graphically represented as a point on the surface of a unit radius Bloch sphere, see Figure 1. In this example, the complementary angle of the latitude, i.e., the colatitude θ is equal to $\pi/6$. The longitude γ is equal to $\pi/2$. A radial line is drawn from the origin to the point on the sphere surface representing the qubit. The point is defined by spherical coordinates. The two probability amplitudes are defined by two angles, namely, θ and γ. Angle θ is the colatitude. Angle γ is the longitude. In the Bloch sphere framework, a qubit is defined by the superposition

$$|\psi\rangle = \cos\frac{\theta}{2}|0\rangle + e^{j\gamma}\sin\frac{\theta}{2}|1\rangle .$$

With θ equal to zero, the qubit is $|0\rangle$. With θ equal to π, the qubit is $|1\rangle$. The Bloch sphere representation highlights the difference between a classical bit and a qubit. A classical bit can only be one of two points on the sphere, the north pole (0) or the south pole (1). A qubit can be any point on the sphere.

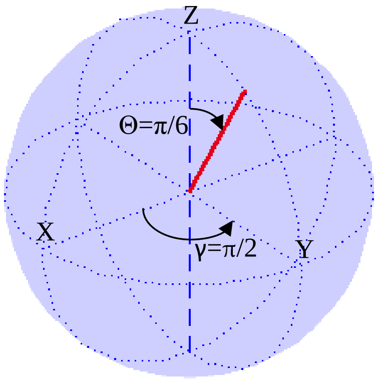

Figure 1. Qbit state modeled by a Bloch sphere (produced using the Hydrogenic Wavefunction Visulization Tool).
