# Supplementary material for: Cyber-physical defense in the quantum Era
Source: Sci Rep. 2022 Feb 3;12:1905. doi: 10.1038/s41598-022-05690-1 (PMC8814167; doi:10.1038/s41598-022-05690-1)
Supplement: Supplementary file 4 — Supplementary Information 4. [file 41598_2022_5690_MOESM4_ESM.pdf]

Appendix D: Q-learning

Q-learning? Reinforcement Learning (RL) builds upon the idea of associating rewards to actions and states. A policy, a function π with domain state set S and co-domain action set A, associates a recommended action to every state. Assuming an agent is following a policy π, every single state $s \in S$ has value $V_\pi(s)$ recursively defined as:

$$V_\pi(s) = \sum_{s' \in S} P_{\pi(s)}(s,s') \cdot \left[R_{\pi(s)}(s,s') + \gamma V_\pi(s')\right]$$

After the execution of the policy determined action $\pi(s)$, s' denotes the successor state of s. $P_{\pi(s)}(s,s')$ represents the probability of s' executing action $\pi(s)$. Under policy π, the evaluation of $V_\pi(s)$ denotes the value of state s. The reward obtained executing action a equal to $\pi(s)$ in state s is $R_a(s,s')$, or $R_{\pi(s)}(s,s')$. Constant γ in $[0,1]$ is a discounting factor, weighting the long-term reward less than the short term one. The goal of RL is to find a policy that makes the agent obtain the best possible reward. Best possible reward is achieved when the world goes through the most valued states. The optimal policy is such that for every state s

$$V_\pi(s) = \max_a \left(\sum_{s' \in S} P_a(s,s') \cdot \left[R_a(s,s') + \gamma V_\pi(s')\right] \right).$$

For obtaining the most rewarding policy, Q-learning uses the concept of Q-value. In reference to a policy π, it is a function Q with domain $S \cdot A$ and co-domain $\mathbb{R}$, defined as

$$Q_\pi(s,a) = \sum_{s' \in S} P_a(s,s') \cdot \left[R_a(s,s') + \gamma V_\pi(s')\right].$$

The optimization is accomplished through a sequence of epochs $t = 0,1,\ldots,n$. The Q-learning algorithm is, at epoch t for the pair (s,a), where $s \in S$ is the current state and $a \in A$ is the executed action,

$$Q_t(s,a) = (1-\alpha)Q_{t-1}(s,a) + \alpha\left[R_a(s,s') + \gamma V_{t-1}(s')\right]$$

with learning factor α in $[0,1]$. For every other action pair (s,a), where $s \in S$ is not the current state or $a \in A$ is not the executed action, $Q_t(s,a)$ is equal to $Q_{t-1}(s,a)$. At epoch $t-1$, the value of state s is

$$V_{t-1}(s) = \max_a Q_{t-1}(s,a)$$

For all pairs (s,a), $Q_0(s,a)$ is set to null. It has been established? that Q_t converges to the optimal policy when n approaches infinity. That is, when t tends to infinity we have that Q_t tends to Q_π with the optimal policy π.
